# The Clinical Efficacy and Safety of Enhanced Recovery After Surgery for Cesarean Section: A Systematic Review and Meta-Analysis of Randomized Controlled Trials and Observational Studies

**DOI:** 10.3389/fmed.2021.694385

**Published:** 2021-08-02

**Authors:** Xianhua Meng, Kai Chen, Chenchen Yang, Hui Li, Xiaohong Wang

**Affiliations:** Department of Obstetrics and Gynecology, Jinan City People's Hospital, Jinan People's Hospital Affiliated to Shandong First Medical University, Shandong, China

**Keywords:** enhanced recovery after surgery, meta-analysis, cesarean section, safety, efficacy

## Abstract

**Background:** Enhanced recovery after surgery (ERAS) has been adopted in some maternity units and studied extensively in cesarean section (CS) in the last years, showing encouraging results in clinic practice. However, the present evidence assessing the effectiveness of ERAS for CS remains weak, and there is a paucity in the published literature, especially in improving maternal outcomes. Our study aimed to systematically evaluate the clinical efficacy and safety of ERAS protocols for CS.

**Methods:** A systematic literature search using Embase, PubMed, and the Cochrane Library was carried out up to October 2020. The appropriate randomized controlled trials (RCTs) and observational studies applying ERAS for patients undergoing CS were included in this study, comparing the effect of ERAS protocols with conventional care on length of hospital stay (LOS), readmission rate, incidence of postoperative complications, postoperative pain score, postoperative opioid use, and cost of hospitalization. All statistical analyses were conducted with the RevMan 5.3 software.

**Results:** Ten studies (four RCTs and six observational studies) involving 16,391 patients were included. ERAS was associated with a decreased LOS (WMD −7.47 h, 95% CI: −8.36 to −6.59 h, *p* < 0.00001) and lower incidence of postoperative complications (RR: 0.50, 95% CI: 0.37 to 0.68, *p* < 0.00001). Moreover, pooled analysis showed that postoperative pain score (WMD: −1.23, 95% CI: −1.32 to −1.15, *p* < 0.00001), opioid use (SMD: −0.46, 95% CI: −0.58 to −0.34, *p* < 0.00001), and hospital cost (SMD:−0.54, 95% CI: −0.63 to −0.45, *p* < 0.00001) were significantly lower in the ERAS group than in the conventional care group. No significant difference was observed with regard to readmission rate (RR: 0.86, 95% CI: 0.48 to 1.54, *p* = 0.62).

**Conclusions:** The available evidence suggested that ERAS applying to CS significantly reduced postoperative complications, lowered the postoperative pain score and opioid use, shortened the hospital stay, and potentially reduced hospital cost without compromising readmission rates. Therefore, protocols implementing ERAS in CS appear to be effective and safe. However, the results should be interpreted with caution owing to the limited number and methodological quality of included studies; hence, future large, well-designed, and better methodological quality studies are needed to enhance the body of evidence.

## Introduction

Cesarean section (CS) is a common operation performed worldwide with approximately 18.5 million procedures being performed annually. Recent global data estimate that nearly 20% of pregnant women give birth *via* cesarean delivery (1). The CS rate has large variations in different countries and regions, ranging from merely 5% in South Sudan to 58.9% in the Dominican Republic (2). In the United States, the cesarean delivery rate is estimated to be almost a third of all births, with over 1.2 million procedures performed every year (3). In China, the CS rate was much higher than the ideal rate recommended by the WHO. More notably, the rate of cesarean delivery on maternal request, the main indication for unnecessary CS, was also high with more than 28% (4). In addition, international analysis shows that the CS rate has witnessed a steady increase and does not show signs of decrease over the past few decades (5). Thus, the huge volume of cesarean deliveries and increasing CS rate has an incremental burden on healthcare systems, leading to higher bed occupancy and financial pressures on the patients and health facilities (6, 7).

While the decrease in the CS rate is crucial, it is also apparent that CS will continue to be a necessary procedure for obstetricians. Therefore, it is not surprising that growing interest will focus on introducing improved perioperative care for CS. In particular, enhanced recovery care is an effective way to improving the clinical and health system benefits of CS, which have been shown to promote rehabilitation and earlier discharge (8). Enhanced recovery after surgery (ERAS) is a multimodal and multidisciplinary approach to optimizing the perioperative management and outcomes (9). The core tenets of the ERAS have been outlined previously and are positioned along the entire surgical care continuum, aiming to alleviate the surgical stress response, promote functional recovery, and achieve rapid recovery (10, 11). ERAS has been widely implemented in multiple surgical disciplines including colorectal, urologic, hepatobiliary, and gynecologic surgery (12, 13). However, the implementation of ERAS in the obstetric field has lagged compared to other surgical subspecialties (14).

Since the ERAS concept was proposed in the field of obstetrics surgery, there has been a slower embrace of ERAS application to CS. Currently, some maternity centers are endeavoring to use ERAS protocol in their clinical practice, showing some advantages over conventional care in CS (15). In the last 5 years, several randomized controlled trials (RCTs) and observational studies were published to evaluate the superiority and feasibility of ERAS for CS, providing better evidence linking ERAS implementation and maternal outcomes including a reduction in hospital stay, lower incidence of complications, and quicker functional recovery (16–19). However, currently no meta-analysis specifically addresses the impact of ERAS on maternal outcomes among women undergoing CS. In this context, quantifying summary evidence involving the comparative effect of ERAS on maternal outcomes is warranted. In this study, we perform a systematic review and meta-analysis to qualitatively and quantitatively assess the clinical efficacy and safety of ERAS protocols for CS, compared with conventional care. Importantly, the overall effect estimated from the existing literature will be helpful in guiding decisions of ERAS practice.

## Methods

### Literature Search and Selection Criteria

This systematic review and meta-analysis were performed in accordance with Preferred Reporting Items for Systematic Review and Meta-analysis (PRISMA) guidelines. A systematic search of the databases of PubMed, Cochrane Library, and Embase was conducted to identify relevant studies. The last search was run on October 19, 2020, and language was limited to English. The following medical subject heading terms and free-text terms individually or in all possible combinations are as follows: “Enhanced recovery after surgery” OR “Accelerated rehabilitation” OR “ERAS” OR “Fast track” OR “Early Recovery” OR “Enhanced Recovery” AND “cesarean” OR “cesarean” OR “cesarean delivery” OR “cesarean section” OR “cesarean section.” In addition, we reviewed the full-text articles designated for inclusion and manually checked the references of the retrieved articles and previous reviews to identify additional eligible studies.

### Inclusion and Exclusion Criteria

Studies meeting the inclusion criteria will be enrolled: (1) participants: patients underwent elective or emergency cesarean section; (2) intervention: ERAS management protocol was proposed in the study; (3) controls: the conventional care protocol was used for patients receiving CS; (4) outcomes: including at least one main outcome of interests; and (5) design: RCTs or observational studies (prospective or retrospective cohort studies).

The exclusion criteria were the following: (I) full text of the article was not available; (II) the outcomes of interest were lacking or impossible to calculate or extrapolate; (III) the types of article were not original articles such as reviews, meta-analyses, comments, case reports, letters to the editor, or protocols; and (IV) republished study.

### Data Extraction and Quality Assessment

Two authors (X. M. and K. C.) screened the titles and abstracts of the initial search results, extracted the data, and assessed for risk of bias independently. Disagreements were resolved by group consensus. Clinical data were extracted from qualified studies and subsequently analyzed. The following information was extracted from the included study: first author, year of publication, country, study design, number of patients enrolled, and patient characteristics (age). The primary outcome was length of hospital stay (LOS). Secondary outcomes were postoperative complications, readmission rate, postoperative pain score, postoperative opioid use, and cost of hospitalization.

The quality of RCTs was assessed by the Cochrane risk of bias tool including selection biases, performance biases, detection biases, attrition biases, reporting biases, and other biases (20). Observational studies were evaluated using the Newcastle–Ottawa Scale (NOS), which included adequacy selection of cohort, comparability of studies, and outcome assessment.

### Statistical Analysis

All statistical analyses were calculated by RevMan 5.3 software. The dichotomous data were performed as risk ratio (RR), and continuous variables were expressed as weighted mean differences (WMD) or standard mean differences (SMD). All results were performed with 95% confidence intervals (CIs). Some data presented with median, and ranges or interquartile ranges were converted into mean and standard deviation using approaches described by Wan et al. (21–23). Initial analyses were assessed using the fixed-effect model. Heterogeneity was tested using *I*^2^ and chi-squared tests. Heterogeneity was categorized as low (*I*^2^ < 50%), moderate (*I*^2^ = 50–75%), and high (*I*^2^ > 75%); *I*^2^ > 50% indicates significant heterogeneity (24). For outcomes detected with significant heterogeneity, sensitivity analyses were conducted to assess the robustness of the results by the sequential omission of individual studies. The sensitivity analyses adopting a random-effect model was also conducted to test the stability of pooled estimates. Publication bias was estimated by the use of funnel plots.

## Results

### Characteristics and Quality of Eligible Studies

**Figure 1:**
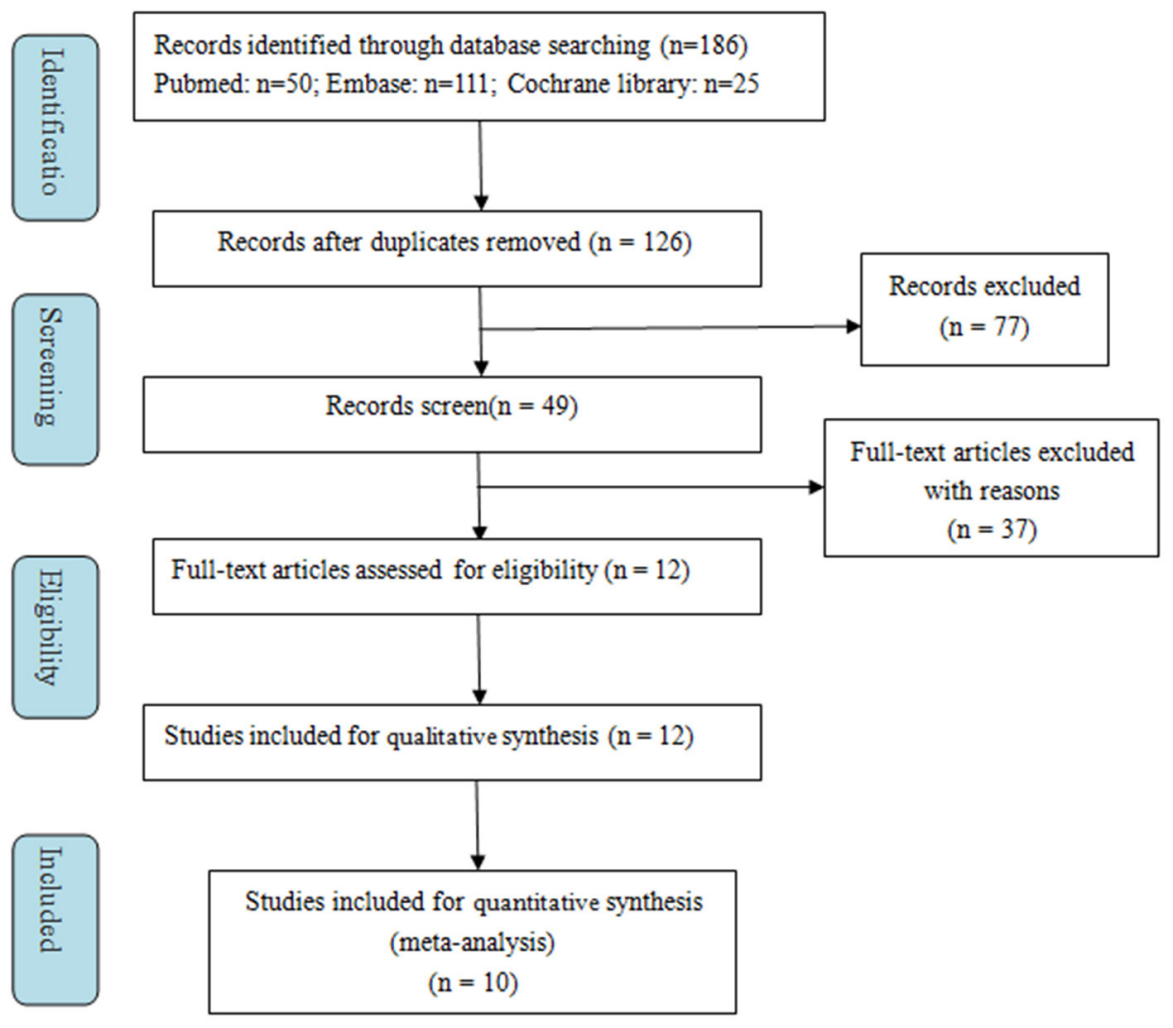
Flowchart indicating the selection process of this meta-analysis.

A total of 186 records were retrieved from the initial literature search. After excluding duplicates (60 records), we identified 126 records to screen titles and abstracts. One hundred fourteen records were removed for various reasons based on the titles and abstracts (reviews, meta-analyses, correction, editorial, response, or supplements, etc.). Subsequently, the remaining 12 full-text articles were assessed for eligibility, and two were excluded for unextractable useful outcomes data. Finally, 10 studies were included in the meta-analysis (16–19, 25–30). The selection process is presented in Figure 1.

**Figure 2:**
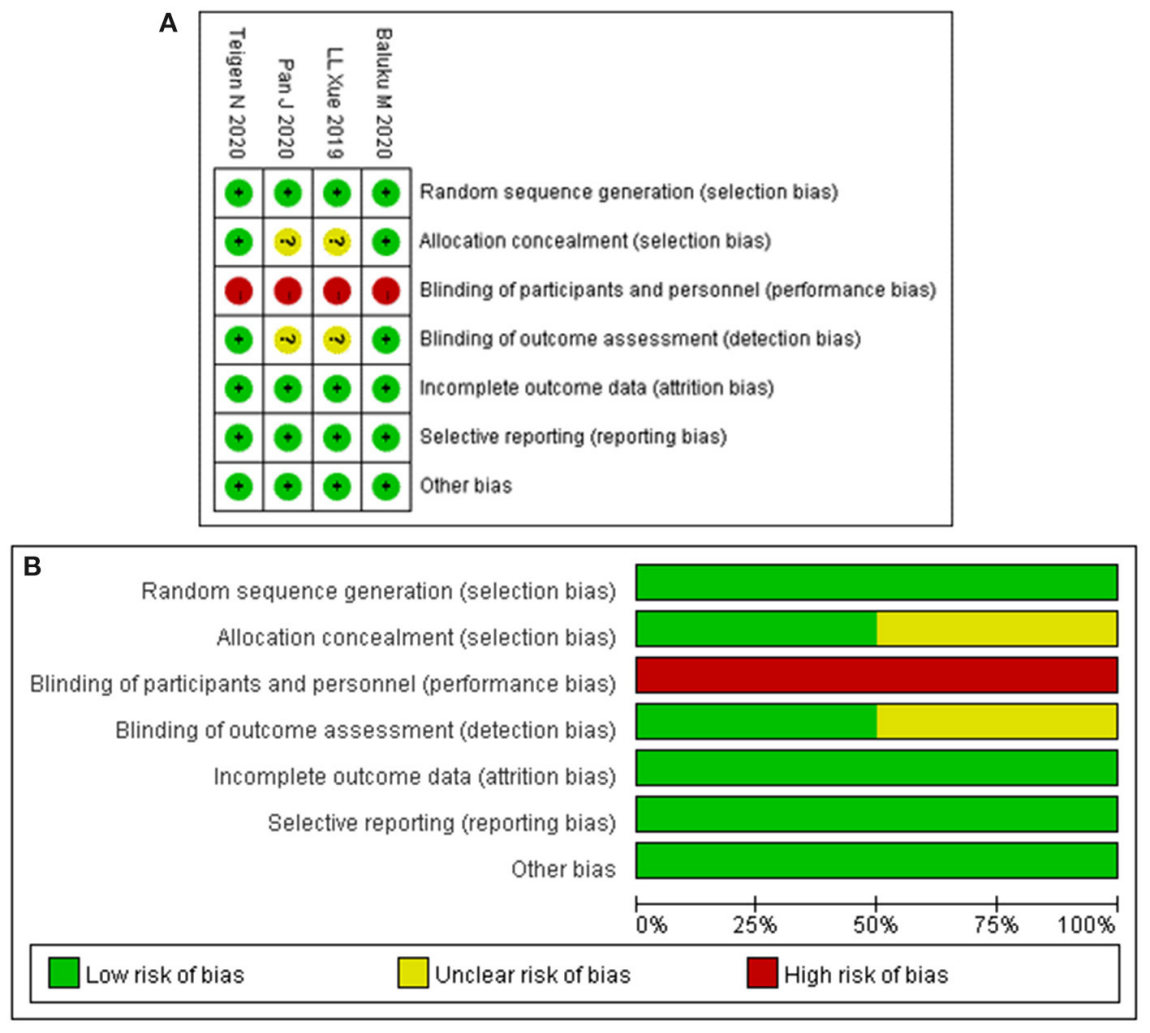
Risk-of-bias analysis: **(A)** risk of bias summary: each risk of bias item for the included study. **(B)** Risk of bias graph: each risk of bias item presented as percentages across all included studies.

Table 1 summarizes the characteristics of the included studies. All studies were published from 2019 to 2020. In total, this meta-analysis involved 16,391 patients, of which 7,595 received ERAS protocols and 8,796 received conventional care. Of the included studies, four were RCTs and six were cohort studies. Among the 10 studies, seven were conducted in America, one in Africa, and two in Asia. Risk of bias for RCTs is shown in Figure 2. Attrition bias, selection bias, detection bias, reporting bias, and other bias were reported adequately in most studies. However, the performance bias was high risk among all the selected studies, because the participants could not be blinded due to the counseling and education of ERAS protocols. The quality assessment of observational studies based on the NOS is presented in Table 2. Only one study achieved the maximum of nine stars. Of the remaining five studies, all achieved eight stars. The majority of bias was found in the comparability of cohorts, because most of the studies lacked a modified control for some important factors.

**Table 1:** Characteristics of included studies.

**Study**	**Year**	**Country/continent**	**Study design**	**ERAS (*n*)**	**Control (*n*)**	**ERAS (age)**	**Control (age)**
Fay EE	2019	USA/North America	Cohort study	531	661	31.9± 5.6	31.6 ±5.5
Pan J	2020	China /Asia	RCT	112	104	33.21 ± 4.49	32.59 ± 4.14
Teigen N	2020	USA/North America	RCT	58	60	30.43 ± 4.92	31.93 ± 5.43
Kleiman AM	2020	USA/North America	Cohort study	160	197	31.0 + 5.2	30.8 + 5.3
Baluku M	2020	Uganda/Africa	RCT	76	77	26.2 ± 5.4	25.1 ± 5.5
Shinnick JK	2020	USA/North America	Cohort study	128	122	31.5	33
Lester SA	2020	USA/North America	Cohort study	112	429	29.79 ± 0.47	30.58 ± 0.28
Hedderson M	2019	USA/North America	Cohort study	4,624	4,689	33.4 ± 5.0	33.3 ± 5.1
Mullman L	2020	USA/North America	Cohort study	1,508	2,171	34	34.1
LL Xue	2019	China /Asia	RCT	286	286	28.91 ± 3.35	28.73 ± 3.09

**Table 2:** Quality assessment of non-randomized studies.

**Study**	**Selection**				**Comparability**	**Outcome**			**Total**
	**1**	**2**	**3**	**4**	**5**	**6**	**7**	**8**	
Fay EE	*	*	*	*	*	*	*	*	8
Kleiman AM	*	*	*	*	*	*	*	*	8
Shinnick JK	*	*	*	*	*	*	*	*	8
Lester SA	*	*	*	*	*	*	*	*	8
Hedderson M	*	*	*	*	*	*	*	*	8
Mullman L	*	*	*	*	**	*	*	*	9

*1. Representativeness of the exposed cohort. 2. Selection of the nonexposed cohort. 3. Ascertainment of exposure. 4. Demonstration that the outcome of interest was not present at start of study. 5. Cohort comparability based on the design or analysis. 6. Outcome assessment. 7. Was follow-up long enough for outcomes to occur? 8. Adequacy of follow-up of cohorts*.

## Outcome Measures

### Length of Hospital Stay

**Figure 3:**
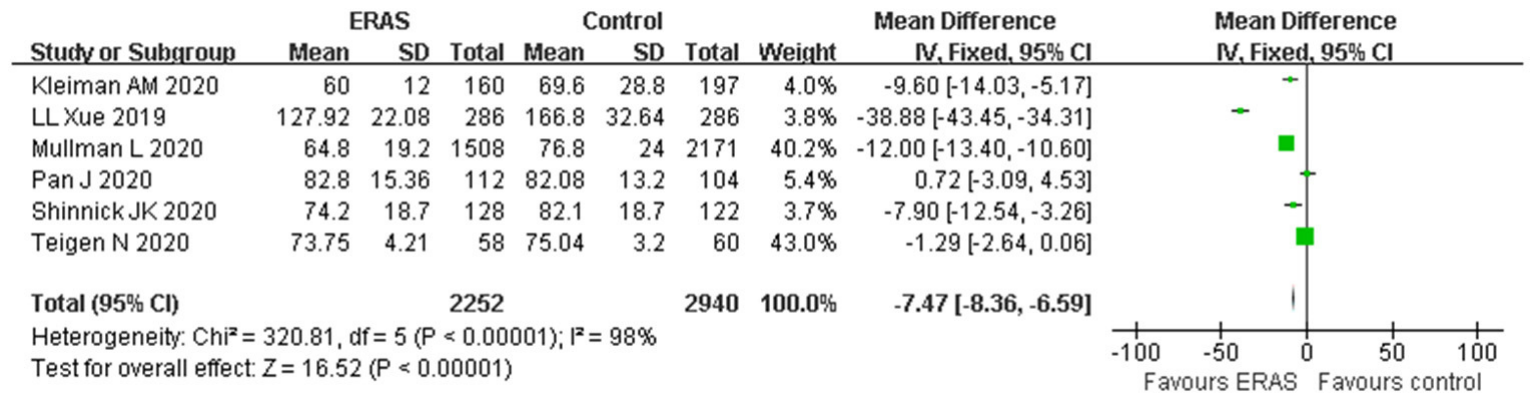
Forest plots assessing the outcomes of length of hospital stay (hours).

A total of six studies with appropriate data reported the LOS. The forest plot indicated that the ERAS protocol was associated with a shorter LOS as compared to the conventional group (WMD −7.47 h, 95% CI: −8.36 to −6.59 h, *p* < 0.00001), with significant heterogeneity (*I*^2^ = 98%, *p* < 0.00001) (Figure 3). The sensitivity analysis showed that the original analysis was not changed by omitting one study in each time, ranging from −6.24 (95% CI, −7.15 to −5.34; *I*^2^ = 97%) to −12.14 (95% CI, −13.31 to −10.96; *I*^2^ = 98%). Furthermore, the result of the random-effect model (WMD −11.38; 95% CI −19.52 to −3.24; *p* < 0.00001) was in agreement with the result of the primary analysis, demonstrating that the result was reasonably stable.

### Postoperative Complications

**Figure 4:**
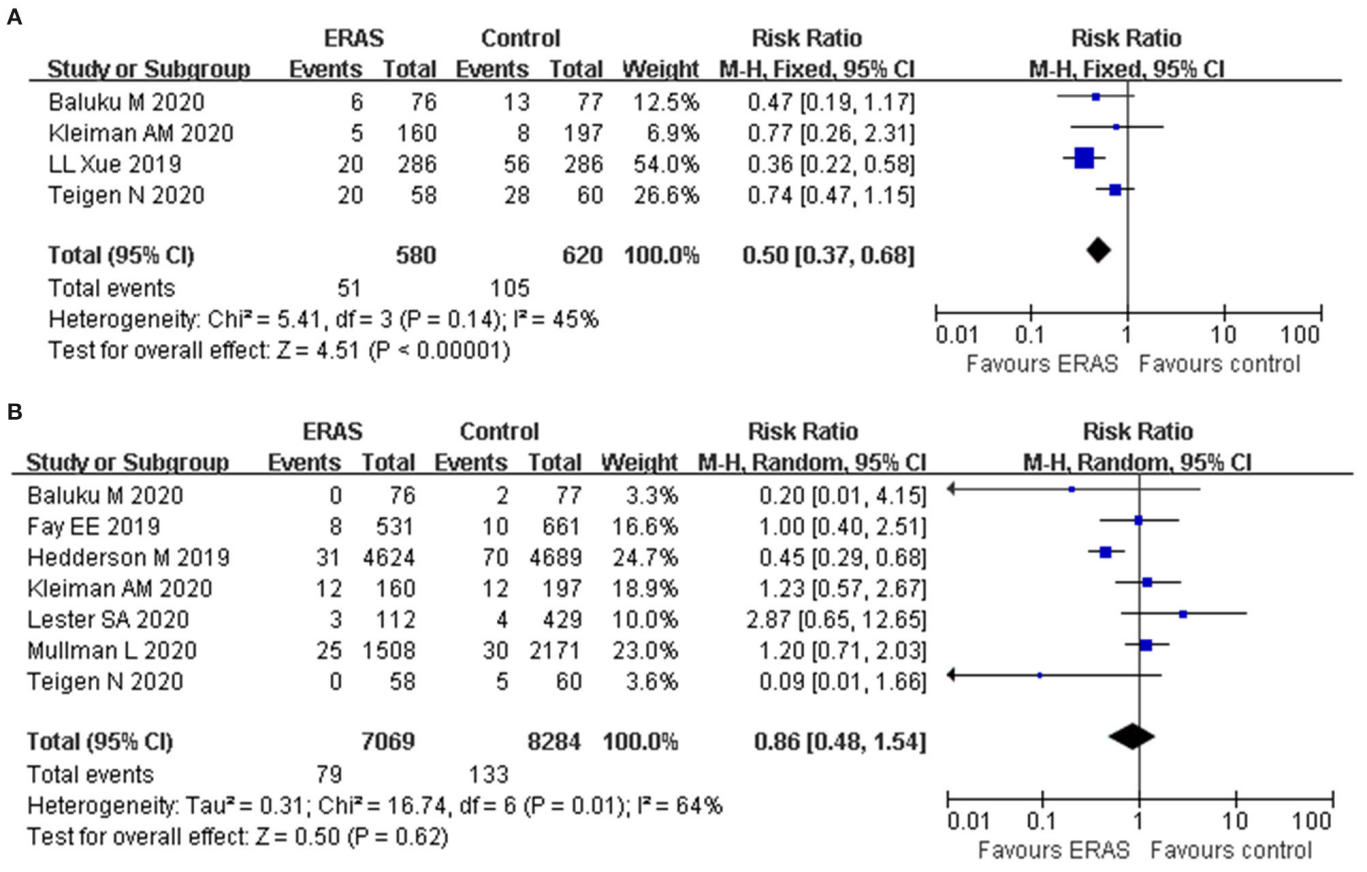
Forest plots with **(A)** postoperative complication and **(B)** readmission rate.

Four studies reported the postoperative complications. The forest plot showed that the rate of postoperative complications was lower in the ERAS group (RR: 0.50, 95% CI: 0.37 to 0.68, *p* < 0.00001, fixed-effect model), without significant heterogeneity (*I*^2^ = 45%, *p* = 0.14) (Figure 4A). In addition, further exclusion of any single study did not materially alter the overall combined RR.

### Readmission Rate

Seven studies reported the readmission rate involving 15,353 participants. The forest plot showed that ERAS decreased the readmission rate in comparison with the conventional group (RR: 0.72, 95% CI: 0.55–0.94, *p* = 0.02, fixed-effect model). The reported heterogeneity was judged to be moderate (*I*^2^ = 64%, *p* = 0.01). However, the pooled data based on the random-effect model identified no significant difference in the readmission rate between the two groups (RR: 0.86, 95% CI 0.48–1.54, *p* = 0.62) (Figure 4B). The sensitivity analysis also confirmed the study by Hedderson et al. which showed a significant effect on heterogeneity (26). The heterogeneity decreased (*I*^2^ = 16%) after removal, as shown in the study by Hedderson et al., and new results also indicated that no significant difference was found in terms of readmission rate (RR: 1.07, 95% CI 0.74–1.53, *p* = 0.73, *I*^2^ = 16%).

### Postoperative Pain Score and Opioid Use

**Figure 5:**
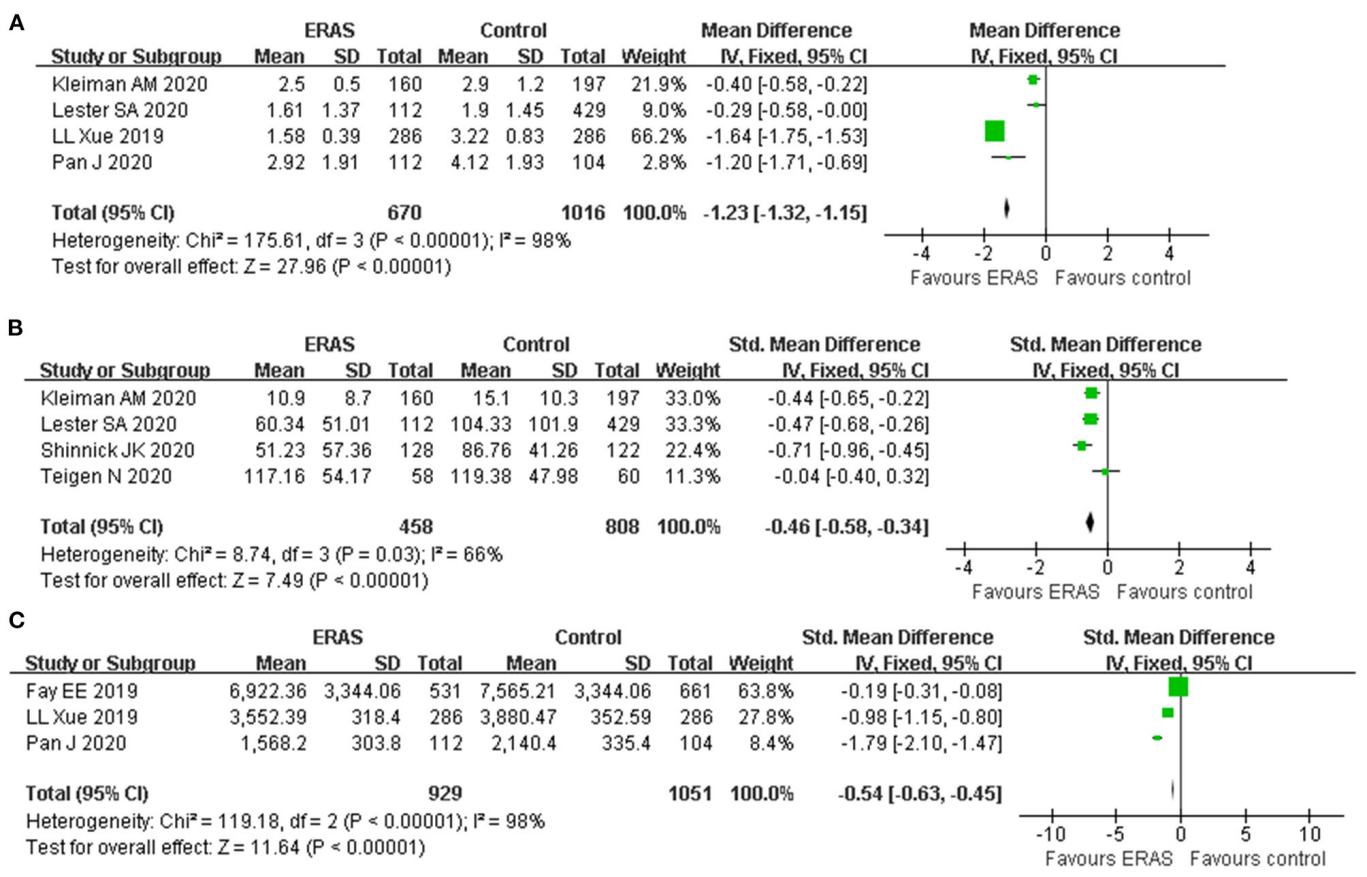
Forest plots with **(A)** postoperative pain score; **(B)** opioid use; and **(C)** cost of hospitalization.

A total of four studies reported a postoperative pain score involving 1,686 participants. The postoperative pain score was significantly lower in patients receiving ERAS than those receiving conventional care (WMD: −1.23, 95% CI: −1.32 to −1.15, *p* < 0.00001, fixed-effect model), with significant heterogeneity (*I*^2^ = 98%, *P* < 0.00001) (Figure 5A). Therefore, the sensitivity analysis showed that the result was stable and no study had a significant impact on the overall results. Also, the random-effect model was applied to this result and showed a similar significant effect (WMD: −0.88, 95% CI: −1.69 to −0.07, *p* = 0.03).

Postoperative opioid use was reported in four studies; pooled results showed that ERAS protocols significantly reduced the postoperative opioid use (SMD: −0.46, 95% CI −0.58 to −0.34, *p* < 0.00001), with moderate heterogeneity (*I*^2^ = 66%, *p* = 0.03) (Figure 5B). Application of a random-effect model revealed a similar significant reduction in opioid use (SMD: −0.44, 95% CI −0.65 to −0.23, *p* < 0.0001, *I*^2^ = 66%).

### Cost of Hospitalization

Only three studies with the appropriate data reported cost of hospitalization involving 10,374 participants. The estimated SMD for the meta-analysis was −0.54 (95% CI −0.63 to −0.45, *p* < 0.00001), indicating a reduction in the hospital cost for the ERAS group as compared with the control group (Figure 5C). The *I*^2^ statistic indicated that there was a significant heterogeneity (*I*^2^ = 98%). Application of the random-effect model did not affect the result (SMD −0.97, 95% CI: −1.78 to −0.16, *p* = 0.02; *I*^2^ = 98%, *p* < 0.00001). Due to the limited included studies, we did not conduct sensitivity analysis by the leave-one-out method for this result.

### Publication Bias and Sensitivity Analysis

Finally, because all pooled analyses include <10 comparative studies fr analysis quantitatively, publication bias detection could not be examined. To evaluate the robustness of the association results, sensitivity analysis using leave-one-out and random-effect modeling was performed to test the stability of the pooled data. Most of the sensitivity analysis results remained stable, indicating the consistency of the pooled results including LOS, postoperative complication, postoperative pain score, postoperative opioid use, and cost of hospitalization. Only the sensitivity analysis for readmission rate showed inconsistent results; we adopted the results of the sensitivity analysis as the final effect.

## Discussion

ERAS is an innovative rehabilitation model applied to the perioperative period in the recent years. It has disrupted the traditional principles of surgical therapeutics and medical understanding with its superior clinical, social, and scientific effects (31). However, the safety and efficacy of ERAS in CS remain controversial. Therefore, we conduct this study to add more granular data regarding the clinical outcomes following ERAS implementation in CS. This is the first meta-analysis including comparative studies of ERAS protocol and conventional care to evaluate the maternal outcomes. Our findings showed that ERAS protocols resulted in favorable outcomes in CS as indicated by reduced postoperative complications, lower hospital costs, and shorter LOS without increasing the need for readmission. It is also worth mentioning that the ERAS protocols reduce the patients' postoperative pain, while not aggravating the opioid use. The sensitivity analyses confirmed the consistency of the results.

LOS is an important index in assessing the benefits of postoperative recovery and has been traditionally one of the key outcomes of ERAS. From the results of the meta-analysis, we found that shorter LOS was presented in the ERAS group. Although the reduction of LOS was <1 day in the analysis, the decreased LOS represented the faster recovery and earlier discharge with clinical significance. Some evidence has documented that early discharge after CS can improve maternal–neonatal bonding and maternal satisfaction coupled with financial savings (32). Moreover, there was also evidence showing that even day-one or day-two discharge appears to be safe and acceptable in low-risk patients undergoing cesarean delivery (33). In the future, the ongoing evaluation of maternal outcomes, neonatal factors, and readmission rates is useful in better confirming optimal discharge times and LOS (34, 35).

Readmission rate is another concern in clinical practice, because higher rate of readmission is a potential barrier for ERAS implementation and negatively affects the life quality of patients (36, 37). Our results did not show that the application of ERAS increases the readmission rate, indicating the safety of applying ERAS in CS. Even our primary results showed that ERAS could decrease the readmission rate; the sensitivity analysis finally confirmed that the readmission rate has no significant difference between two groups. Admittedly, postoperative complication is also a major concern in postoperative care, because postoperative complications hindered early discharge and potentially increased the healthcare costs and utilization of hospital resources. Our results suggested that ERAS protocol reduced the rate of postoperative complications. This result was consistent with implementation of ERAS in other non-obstetric patients, demonstrating the superiority of ERAS in improvement for postoperative complications.

There are several reasons addressing ERAS implementation with such striking clinical results. Firstly, detailed preoperative education and psychological counseling from ERAS protocols will be helpful in easing the psychological pressure and improving patient compliance to ERAS protocol (38). Secondly, ERAS protocols reduce fasting time and increase carbohydrate intake to relieve the stress of hunger and anxiety before CS, decreasing the insulin resistance and the loss of nutrition in the postoperative period (39). Third, ERAS protocols advocate early removal of urinary catheter and mobilization, thereby decreasing the risk of urinary tract infections and postoperative venous thromboembolism (VTE) (40–42). Fourth, standardized care practices, standardization of the use of prophylactic antibiotics, and early mobilization in ERAS have supported significant reductions in postoperative infections such as surgical site infections, lung infection, and urinary tract infection (43). Fifth, excellent analgesia, intraoperative warming, and early postoperative oral feeding are all essential to accelerating recovery *via* maintaining body homeostasis, promoting discharge earlier, and reducing postoperative complications (44). More importantly, ERAS works improve most parts of the perioperative process and achieve additive benefits beyond the individual modifications (45).

Postoperative pain management has been an essential issue related to postoperative recovery in patients receiving CS (34, 46). While opioid use is an important aspect of postoperative pain control in patients undergoing CS, excessive opioid use brings many side effects affecting maternal and newborn health (47, 48). Additionally, postpartum pain and opioid-related side effects may influence the maternal–fetal bonding and maternal recovery (28). In this regard, to test the evidence considering the connections between implementation of ERAS in CS and postoperative pain and opioid use is meaningful. Our pooled analysis supported that ERAS implementation improves the postoperative pain scores and lowers the opioid consumption in CS. Interestingly, Hedderson et al. showed that women undergoing CS and receiving ERAS protocol had higher acceptable pain scores for all postoperative days (26). We speculate that beneficial effects to pain control and opioid consumption arising from ERAS may be multifactorial. An important aspect of ERAS is the multimodal analgesia scheme, which has shown to alleviate concerns regarding opioid use, reduce pain scores, and enhance patient comfort (49). In particular, the multimodal approach to opioid-sparing pain control adopted in ERAS also brought other benefits such as earlier recovery of gastrointestinal function, early ambulation, fetal protection, and reduced risk of maternal opioid abuse (50, 51). Importantly, the decrease in postoperative pain score does not come at the price of increased opioid consumption. Therefore, it could be inferred that pain relief mainly benefited from the effect of ERAS protocol implementation rather than excessive use of opioids. Our results highlight the importance of ERAS for post-CS pain management.

In addition, other factors may influence pain relief including anxiety and anticipated pain (52). Improving these concerns before surgery may reduce pain perception and increase maternal satisfaction. Perioperative education and psychological counseling of ERAS, anticipated pain severity, and pain control strategies obviously reduce anxiety and set realistic expectations, positively influencing the opioid consumption and pain scores (27, 53). Taken together, the use of the multimodal analgesia approach, characteristic of ERAS, might be a viable strategy for lowering opioid use and pain score postoperatively.

Economic burden is an unignored factor considered in the clinical practice of ERAS. Our results showed that the cost of hospitalization was significantly lower in the ERAS group than in the control group, suggesting that implementing ERAS protocol in CS is cost-effective. However, due to the limited studies assessing data of hospital cost, more high-quality trials were needed to determine the true cost-effectiveness of ERAS. We speculate that the saving of hospital costs mainly benefits from shorter hospital stay, reduced drugs, and lower complication rate, although ERAS contains the use of diverse medical care modalities and treatment approaches (17). The present encouraging results would greatly promote the implementation of ERAS protocols in maternity units.

In addition to aforementioned advantages of ERAS implementation in CS, it may have other potential benefits such as improving patient satisfaction and increasing the breastfeeding rate. However, data involving these specific effects of ERAS are limited and unclear. Only a few investigations demonstrated that ERAS may improve patient satisfaction, breastfeeding, and mother–child bonding. Hopefully, more studies will be conducted to evaluate these beneficial effects of ERAS, further exploring the effects referring to neonate outcome, postpartum depression, and service efficiency.

Despite our careful work on the currently available evidence, several limitations should be interpreted in this meta-analysis. Firstly, only four RCTs were included in the present study and some outcomes mainly derived from cohort studies rather than RCTs; thus, the results may have been influenced by information bias, selection bias, and detection bias, as well as confounding bias. Secondly, none of the RCTs featured blinding, potentially leading to performance bias and measurement bias. It is noted that the blinding for the ERAS protocol is not feasible in clinical practice. Thirdly, the ERAS protocol elements in each study may be different slightly, leading to inescapable heterogeneity.

## Conclusions

Our results showed that protocols implementing ERAS in CS could shorten LOS and hospital cost and reduce the incidence of complications, postoperative pain score, and opioid use, but could not increase the rates of readmission. Our data add to the evidence supporting that ERAS protocols applied to CS are feasible, effective, and safe. However, limited to the quantity and quality of the studies and their potential heterogeneity, further large and randomized controlled studies should be undertaken to confirm the present findings.

## Data Availability Statement

The original contributions presented in the study are included in the article/supplementary material, further inquiries can be directed to the corresponding author/s.

## Author Contributions

XM, KC, and XW designed the research and the study concept. XM and KC performed the data extraction. XM, KC, and CY analyzed and interpreted the data. XM, KC, and HL performed the quality and risk assessment. XM, KC, CY, HL, and XW wrote the article. All the coauthors granted final approval of the version of this article to be published.

## Conflict of Interest

The authors declare that the research was conducted in the absence of any commercial or financial relationships that could be construed as a potential conflict of interest.

## Publisher's Note

All claims expressed in this article are solely those of the authors and do not necessarily represent those of their affiliated organizations, or those of the publisher, the editors and the reviewers. Any product that may be evaluated in this article, or claim that may be made by its manufacturer, is not guaranteed or endorsed by the publisher.

## References

[1] Betrán AP Ye J Moller AB Zhang J Gülmezoglu AM Torloni MR. The increasing trend in cesarean section rates: global, regional and national estimates: 1990-2014. PLoS One. (2016) 11:e0148343. 10.1371/journal.pone.0148343 26849801 PMC4743929

[2] Boatin AA Schlotheuber A Betran AP Moller AB Barros AJD Boerma T. Within country inequalities in cesarean section rates: observational study of 72 low and middle income countries. BMJ (Clinical research ed). (2018) 360:k55. 10.1136/bmj.k55 29367432 PMC5782376

[3] Martin JA Hamilton BE Osterman MJK Driscoll AK Drake P. Births: final data for 2016. Natl Vital Stat Rep. (2018) 67:1–55. 29775434

[4] Yu Y Zhang X Sun C Zhou H Zhang Q Chen C. Reducing the rate of cesarean delivery on maternal request through institutional and policy interventions in Wenzhou, China. PLoS ONE. (2017) 12:e0186304. 10.1371/journal.pone.0186304 29155824 PMC5695783

[5] Boerma T Ronsmans C Melesse DY Barros AJD Barros FC Juan L. Global epidemiology of use of and disparities in cesarean sections. Lancet (London, England). (2018) 392:1341–8. 10.1016/S0140-6736(18)31928-7 30322584

[6] Halder S Onwere C Singh N Cox M Yentis SM. Cost reduction and increased patient satisfaction with enhanced recovery for elective cesarean section. Int J Obstet Anesth. (2014) 23:S25. 10.1016/j.ijoa.2014.03.011

[7] Roy S Montgomery Irvine L. Cesarean section rate and postnatal bed occupancy: a retrospective study replacing assumptions with evidence. BMC Health Serv Res. (2018) 18:760. 10.1186/s12913-018-3570-3 30290798 PMC6173897

[8] Corso E Hind D Beever D Fuller G Wilson MJ Wrench IJ. Enhanced recovery after elective cesarean: a rapid review of clinical protocols, and an umbrella review of systematic reviews. BMC Pregnancy Childbirth. (2017) 17:91. 10.1186/s12884-017-1265-0 28320342 PMC5359888

[9] Kehlet H Wilmore DW. Evidence-based surgical care and the evolution of fast-track surgery. Ann Surg. (2008) 248:189–98. 10.1097/SLA.0b013e31817f2c1a 18650627

[10] Sun YM Wang Y Mao YX Wang W. The safety and feasibility of enhanced recovery after surgery in patients undergoing pancreaticoduodenectomy: an updated meta-analysis. Biomed Res Int. (2020) 2020:7401276. 10.1155/2020/7401276 32462014 PMC7232716

[11] Rawlinson A Kang P Evans J Khanna A. A systematic review of enhanced recovery protocols in colorectal surgery. Ann R Coll Surg Engl. (2011) 93:583–8. 10.1308/147870811X605219 22041232 PMC3566681

[12] Melnyk M Casey RG Black P Koupparis AJ. Enhanced recovery after surgery (ERAS) protocols: Time to change practice? Can Urol Assoc J. (2011) 5:342–8. 10.5489/cuaj.11002 22031616 PMC3202008

[13] Elias KM. Understanding Enhanced recovery after surgery guidelines: an introductory approach. J Laparoendosc Adv Surg Tech A. (2017) 27:871–5. 10.1089/lap.2017.0342 28704133

[14] Suharwardy S Carvalho B. Enhanced recovery after surgery for cesarean delivery. Curr Opin Obstet Gynecol. (2020) 32:113–20. 10.1097/GCO.0000000000000616 32068543

[15] Wrench IJ Allison A Galimberti A Radley S Wilson MJ. Introduction of enhanced recovery for elective cesarean section enabling next day discharge: a tertiary center experience. Int J Obstet Anesth. (2015) 24:124–30. 10.1016/j.ijoa.2015.01.003 25794417

[16] Baluku M Bajunirwe F Ngonzi J Kiwanuka J Ttendo SA. Randomized controlled trial of enhanced recovery after surgery versus standard of care recovery for emergency cesarean deliveries at mbarara hospital, Uganda. Anesth Analg. (2020) 130:769–76. 10.1213/ANE.0000000000004495 31663962

[17] Pan J Hei Z Li L Zhu D Hou H Wu H. The advantage of implementation of enhanced recovery after surgery (ERAS) in acute pain management during elective cesarean delivery: A prospective randomized controlled trial. Ther Clin Risk Manag. (2020) 16:369–78. 10.2147/TCRM.S244039 32440135 PMC7210449

[18] Teigen NC Sahasrabudhe N Doulaveris G Xie X Negassa A Bernstein J. Enhanced recovery after surgery at cesarean delivery to reduce postoperative length of stay: a randomized controlled trial. Am J Obstet Gynecol. (2020) 222:372. 10.1016/j.ajog.2019.10.009 31669738

[19] Xue LL Zhang JZ Shen HX Hou Y Ai L Cui XM. [The application of rapid rehabilitation model of multidisciplinary cooperation in cesarean section and the evaluation of health economics]. Zhonghua yi xue za zhi. (2019) 99:3335–9. 10.3760/cma.j.issn.0376-2491.2019.42.012 31715671

[20] Higgins JP Altman DG Gøtzsche PC Jüni P Moher D Oxman AD. The Cochrane Collaboration's tool for assessing risk of bias in randomized trials. BMJ (Clinical research ed). (2011) 343:d5928. 10.1136/bmj.d5928 22008217 PMC3196245

[21] Luo D Wan X Liu J Tong T. Optimally estimating the sample mean from the sample size, median, mid-range, and/or mid-quartile range. Stat Methods Med Res. (2018) 27:1785–805. 10.1177/0962280216669183 27683581

[22] Wan X Wang W Liu J Tong T. Estimating the sample mean and standard deviation from the sample size, median, range and/or interquartile range. BMC Med Res Methodol. (2014) 14:135. 10.1186/1471-2288-14-135 25524443 PMC4383202

[23] Hozo SP Djulbegovic B Hozo I. Estimating the mean and variance from the median, range, and the size of a sample. BMC Med Res Methodol. (2005) 5:13. 10.1186/1471-2288-5-13 15840177 PMC1097734

[24] Higgins JP Thompson SG Deeks JJ Altman DG. Measuring inconsistency in meta-analyses. BMJ (Clinical research ed). (2003) 327:557–60. 10.1136/bmj.327.7414.557 PMC192859 12958120

[25] Fay EE Hitti JE Delgado CM Savitsky LM Mills EB Slater JL. An enhanced recovery after surgery pathway for cesarean delivery decreases hospital stay and cost. Am J Obstet Gynecol. (2019) 221:349. 10.1016/j.ajog.2019.06.041 31238038

[26] Hedderson M Lee D Hunt E Lee K Xu F Mustille A. Enhanced recovery after surgery to change process measures and reduce opioid use after cesarean delivery: a quality improvement initiative. Obstet Gynecol. (2019) 134:511–9. 10.1097/AOG.0000000000003406 31403591 PMC7282661

[27] Kleiman AM Chisholm CA Dixon AJ Sariosek BM Thiele RH Hedrick TL. Evaluation of the impact of enhanced recovery after surgery protocol implementation on maternal outcomes following elective cesarean delivery. Int J Obstet Anesth. (2020) 43:39–46. 10.1016/j.ijoa.2019.08.004 31522935

[28] Lester SA Kim B Tubinis MD Morgan CJ Powell MF. Impact of an enhanced recovery program for cesarean delivery on postoperative opioid use. Int J Obstet Anesth. (2020) 43:47–55. 10.1016/j.ijoa.2020.01.005 32044216

[29] Mullman L Hilden P Goral J Gwacham N Tauro C Spinola K. Improved outcomes with an enhanced recovery approach to cesarean delivery. Obstet Gynecol. (2020) 136:685–91. 10.1097/AOG.0000000000004023 32925620 PMC7505153

[30] Shinnick JK Ruhotina M Has P Kelly BJ Brousseau EC O'Brien J. Enhanced recovery after surgery for cesarean delivery decreases length of hospital stay and opioid consumption: a quality improvement initiative. Am J Perinatol. (2020). [Epub ahead of print]. 10.1055/s-0040-1709456 32485757

[31] Ljungqvist O Scott M Fearon KC. Enhanced recovery after surgery: a review. JAMA Surg. (2017) 152:292–8. 10.1001/jamasurg.2016.4952 28097305

[32] Bowden SJ Dooley W Hanrahan J Kanu C Halder S Cormack C. Fast-track pathway for elective cesarean section: a quality improvement initiative to promote day 1 discharge. BMJ open quality. (2019) 8:e000465. 10.1136/bmjoq-2018-000465 31259280 PMC6567941

[33] Sultan P Sharawi N Blake L Carvalho B. Enhanced recovery after cesarean delivery versus standard care studies: a systematic review of interventions and outcomes. Int J Obstet Anesth. (2020) 43:72–86. 10.1016/j.ijoa.2020.03.003 32299662

[34] Benhamou D Kfoury T. Enhanced recovery after cesarean delivery: Potent analgesia and adequate practice patterns are at the heart of successful management. Anaesth Crit Care Pain Med. (2016) 35:373–5. 10.1016/j.accpm.2016.11.001 27989284

[35] Prior E Santhakumaran S Gale C Philipps LH Modi N Hyde MJ. Breastfeeding after cesarean delivery: a systematic review and meta-analysis of world literature. Am J Clin Nutr. (2012) 95:1113–35. 10.3945/ajcn.111.030254 22456657

[36] Khorgami Z Andalib A Aminian A Kroh MD Schauer PR Brethauer SA. Predictors of readmission after laparoscopic gastric bypass and sleeve gastrectomy: a comparative analysis of ACS-NSQIP database. Surg Endosc. (2016) 30:2342–50. 10.1007/s00464-015-4477-2 26307598

[37] Lyon A Solomon MJ Harrison JD. A qualitative study assessing the barriers to implementation of enhanced recovery after surgery. World J Surg. (2014) 38:1374–80. 10.1007/s00268-013-2441-7 24385194

[38] Wilson RD Caughey AB Wood SL Macones GA Wrench IJ Huang J. Guidelines for antenatal and preoperative care in cesarean delivery: enhanced recovery after surgery society recommendations (Part 1). Am J Obstet Gynecol. (2018) 219:523. 10.1016/j.ajog.2018.09.015 30118692

[39] Nygren J Thacker J Carli F Fearon KC Norderval S Lobo DN. Guidelines for perioperative care in elective rectal/pelvic surgery: Enhanced Recovery After Surgery (ERAS(®)) Society recommendations. World J Surg. (2013) 37:285–305. 10.1007/s00268-012-1787-6 23052796

[40] D'Alton ME Friedman AM Smiley RM Montgomery DM Paidas MJ D'Oria R. National partnership for maternal safety: consensus bundle on venous thromboembolism. Anesth Analg. (2016) 123:942–9. 10.1213/ANE.0000000000001569 27636577

[41] van der Leeden M Huijsmans R Geleijn E. de Lange-de Klerk ES, Dekker J, Bonjer HJ, et al. Early enforced mobilization following surgery for gastrointestinal cancer: feasibility and outcomes. Physiotherapy. (2016) 102:103–10. 10.1016/j.physio.2015.03.3722 26059985

[42] Peahl AF Kountanis JA Smith RD. Postoperative urinary catheter removal for enhanced recovery after cesarean protocols. Am J Obstet Gynecol. (2020) 222:634. 10.1016/j.ajog.2020.01.040 31981514

[43] Peahl AF Smith R Johnson TRB Morgan DM Pearlman MD. Better late than never: why obstetricians must implement enhanced recovery after cesarean. Am J Obstet Gynecol. (2019) 221:117. 10.1016/j.ajog.2019.04.030 31055033

[44] Aluri S Wrench IJ. Enhanced recovery from obstetric surgery: a UK survey of practice. Int J Obstet Anesth. (2014) 23:157–60. 10.1016/j.ijoa.2013.11.006 24631055

[45] Lumb AB McLure HA. AAGBI recommendations for standards of monitoring during anesthesia and recovery 2015 - a further example of ‘aggregation of marginal gains’. Anesthesia. (2016) 71:3–6. 10.1111/anae.13327 26583810

[46] Wu CL Rowlingson AJ Partin AW Kalish MA Courpas GE Walsh PC. Correlation of postoperative pain to quality of recovery in the immediate postoperative period. Reg Anesth Pain Med. (2005) 30:516–22. 10.1097/00115550-200511000-00003 16326335

[47] Eisenach JC Pan PH Smiley R Lavand'homme P Landau R Houle TT. Severity of acute pain after childbirth, but not type of delivery, predicts persistent pain and postpartum depression. Pain. (2008) 140:87–94. 10.1016/j.pain.2008.07.011 18818022 PMC2605246

[48] Carvalho B Butwick AJ. Post cesarean delivery analgesia. Best Pract Res Clin Anaesthesiol. (2017) 31:69–79. 10.1016/j.bpa.2017.01.003 28625307

[49] Sutton CD Carvalho B. Optimal pain management after cesarean delivery. Anesthesiol Clin. (2017) 35:107–24. 10.1016/j.anclin.2016.09.010 28131114

[50] ACOG Committee Opinion No. 742. Postpartum pain management. Obstetr Gynecol. (2018) 132:e35–e43. 10.1097/AOG.0000000000002683 29781876

[51] Stone AB Wick EC Wu CL Grant MC. The US opioid crisis: a role for enhanced recovery after surgery. Anesth Analg. (2017) 125:1803–5. 10.1213/ANE.0000000000002236 28678072

[52] Pan PH Tonidandel AM Aschenbrenner CA Houle TT Harris LC Eisenach JC. Predicting acute pain after cesarean delivery using three simple questions. Anesthesiology. (2013) 118:1170–9. 10.1097/ALN.0b013e31828e156f 23485992 PMC3951732

[53] Stamenkovic DM Rancic NK Latas MB Neskovic V Rondovic GM Wu JD. Preoperative anxiety and implications on postoperative recovery: what can we do to change our history. Minerva Anestesiol. (2018) 84:1307–17. 10.23736/S0375-9393.18.12520-X 29624026

